# Pharmacological modulation of the gut-brain axis: psychobiotics in focus for depression therapy

**DOI:** 10.3389/fphar.2025.1665419

**Published:** 2025-09-26

**Authors:** Zakirah Zainal Abidin, Zaw Myo Hein, Che Mohd Nasril Che Mohd Nassir, Norshafarina Shari, Muhammad Danial Che Ramli

**Affiliations:** ^1^ School of Graduate Studies, Postgraduate Centre, Management and Science University, Shah Alam, Selangor, Malaysia; ^2^ Department of Basic Medical Sciences, College of Medicine, Ajman University, Ajman, United Arab Emirates; ^3^ Department of Anatomy and Physiology, School of Basic Medical Sciences, Faculty of Medicine, Universiti Sultan Zainal Abidin, Kuala Terengganu, Terengganu, Malaysia; ^4^ Faculty of Health and Life Sciences, Management and Science University, Shah Alam, Selangor, Malaysia

**Keywords:** major depressive disorder, therapy, psychobiotics, postbiotics, gut microbiota

## Abstract

Major depressive disorder (MDD) is a multifactorial condition shaped by neurobiological, psychological, and environmental influences. Recent evidence highlights the gut–brain axis (GBA), a bidirectional communication system linking the gastrointestinal tract and central nervous system, as an important contributor to MDD pathogenesis via microbiota-mediated mechanisms. This narrative review synthesizes findings from preclinical and clinical studies published in the last decade, with emphasis on mechanistic insights from animal models and translational data from human cohorts. Key pathways include the microbial regulation of neurotransmitter production, immune modulation, vagus nerve signalling, and the metabolism of short-chain fatty acids (SCFAs). Dysbiosis in MDD is frequently characterized by reductions in butyrate-producing genera and elevations in pro-inflammatory taxa which have been linked to neuroinflammation, impaired neurotransmitter synthesis, and hypothalamic-pituitary-adrenal (HPA) axis dysregulation. Interventions such as probiotics, prebiotics, synbiotics, and psychobiotics show promise in alleviating depressive symptoms by modulating the gut microbiota. Emerging evidence also supports the beneficial roles of postbiotics, non-viable microbial products with immunomodulatory and neuroactive potential. Overall, microbial modulation offers a novel adjunctive strategy for depression management, particularly in treatment-resistant cases or to reduce the side effects of conventional drugs. However, heterogeneity in study design, small sample sizes, and limited causal evidence underscore the need for rigorous, large-scale trials. Future directions should prioritize identification of microbial biomarkers, optimization of strain-specific and dose–response data, and integration of gut-targeted approaches into personalized mental healthcare.

## 1 Introduction

Major depressive disorder (MDD) is a prevalent and disabling mental health condition, characterized by persistent low mood, anhedonia, cognitive impairments, and disruptions in sleep, appetite, and energy regulation lasting for a minimum of 2 weeks (Otte et al., 2016). With an estimated global prevalence affecting over 320 million individuals, MDD represents a leading cause of disability worldwide. The World Health Organization (WHO) anticipates it will become the foremost contributor to the global burden of disease in the near future (Nik Ramli et al., 2024; Zainal Abidin et al., 2023). Unlike transient experiences of sadness or grief, depression is marked by chronicity and functional impairment, often persisting long after triggering events have passed and significantly affecting quality of life and social participation (Belmaker and Agam, 2008).

The pathogenesis of depression is multifactorial, resulting from the intricate interaction of biological, psychological, and environmental influences. Neurobiological research has highlighted structural and functional changes in brain regions involved in emotion and cognition, particularly the prefrontal cortex (PFC), hippocampus, and amygdala (Sun et al., 2023). Neuroimaging consistently reveals reduced volume and hypoactivity in these areas among individuals with depression, impairing executive functioning and memory (Zhang et al., 2018). Concurrently, hyperactivation of the hypothalamic-pituitary-adrenal (HPA) axis and elevated cortisol levels contribute to neuronal atrophy and symptom exacerbation (Jesulola et al., 2018; Rafiee Sardooi et al., 2020). In addition to monoaminergic deficits, newer evidence implicates dysregulation in glutamate and gamma-aminobutyric acid (GABA) pathways both vital to synaptic plasticity and emotional stability (Sarawagi et al., 2021).

Moreover, psychosocial stressors, such as trauma, chronic stress, and limited social support, further intensify vulnerability to depression, particularly among individuals with genetic predispositions. This gene-environment interaction reinforces the individualized and dynamic nature of MDD’s etiology and progression (Belmaker and Agam, 2008).

While traditional treatments target central nervous system (CNS) dysfunction using pharmacological and psychotherapeutic modalities, growing attention has turned toward peripheral contributors to mental health, most notably the gut microbiota. The gut-brain axis (GBA), a bidirectional communication system connecting the gastrointestinal tract with the CNS, has emerged as a critical regulator of mood, cognition, and behavior (Carabotti et al., 2015; Morys et al., 2024).

Emerging research suggests that gut microbiota composition shaped by diet, environment, age, sex, and genetic factors can significantly impact neurophysiological and immune pathways relevant to depression (Ullah et al., 2024). Germ-free animal models have demonstrated altered brain structure, behavior, and emotional regulation in the absence of a functional microbiome, reinforcing its developmental importance (Nakhal et al., 2024). Certain microbial species contribute directly to the synthesis of key neurotransmitters, including serotonin and GABA, while also regulating inflammation via cytokine signaling processes increasingly recognized as central to MDD pathophysiology (Foster and McVey Neufeld, 2013; Irum et al., 2023).

Crucially, the gut microbiota is more amenable to modulation than central neural pathways. Interventions such as dietary adjustments, probiotic and prebiotic supplementation, and fecal microbiota transplantation (FMT) represent accessible, non-invasive strategies for restoring microbial balance and potentially mitigating depressive symptoms (Zhang et al., 2025). These emerging approaches offer promising adjuncts or alternatives to conventional antidepressant therapy and underscore the need for more integrative mental healthcare frameworks.

Thus, in this review, we examine the mechanisms linking gut microbiota dysbiosis to MDD and evaluate the therapeutic potential of microbiota-targeted strategies including probiotics, prebiotics, synbiotics, dietary interventions, FMT, and gut-focused pharmacological agents. We also discuss the translational potential of these findings for health promotion and behavioral health interventions, particularly in the context of education, prevention, and community-level mental health strategies.

### 1.1 Search and selection

This review is a narrative synthesis, not a systematic review. The literature was mainly found through PubMed indexed articles published between 2013 and 2025 using the terms “gut microbiota,” “major depressive disorder,” psychobiotics,” “probiotics,” “prebiotics,” “synbiotics,” and “postbiotics.” Only publications in English were assessed. Preclinical data was separated from clinical evidence based on study designs, with randomised controlled trials and cohort studies involving humans labelled clinical studies, while animal models and *in vitro* studies were deemed preclinical.

## 2 Gut microbiota system

The gut microbiota plays a fundamental role in maintaining human health, influencing a wide range of physiological processes including hormone production, immune regulation, metabolism, and communication between the gut and the brain. Central to this dynamic interaction is the GBA, a complex bidirectional communication network that links the gastrointestinal tract and the CNS via neural, endocrine, immune, and metabolic pathways (Ullah et al., 2024).

This sophisticated system facilitates constant crosstalk between the gut microbiota and the brain, allowing microbial signals to influence cognition, emotion, and behavior. Conversely, psychological stress and neural activity can impact gut function and microbiota composition, illustrating the deeply interconnected nature of this axis. Dysbiosis (e.g., reduced *Faecalibacterium*, increased *Eggerthella*) has been linked to systemic inflammation, immune dysregulation, and neuropsychiatric conditions, including MDD, with implications for drug metabolism and treatment response (Foster and McVey Neufeld, 2013; Cheng et al., 2024).

The gut microbiome is a diverse ecosystem composed of bacteria, viruses, archaea, and eukaryotic microorganisms, with a collective gene pool that vastly exceeds that of the human host. It harbors approximately 1,000 bacterial species and 7,000 strains. Among the dominant phyla are *Firmicutes* (e.g., *Lactobacillus*, *Eubacterium*, *Clostridium*), *Bacteroidetes* (e.g., *Bacteroides*, *Prevotella*), *Actinobacteria*, *Proteobacteria*, and *Verrucomicrobia*, all of which play essential roles in metabolic and neuroimmune homeostasis (Ullah et al., 2024).

The gut microbiota communicates with the enteric nervous system (ENS) often referred to as the “second brain” and the CNS by producing key signaling molecules such as cytokines, SCFAs, and neurotransmitter precursors. These biochemical messengers can influence blood-brain barrier (BBB) integrity, modulate inflammatory pathways, and alter the synthesis of mood-related neurotransmitters such as serotonin and GABA (Ullah et al., 2024).

Disruptions in the GBA due to gut dysbiosis can impair these communication mechanisms, leading to increased systemic inflammation and altered neurotransmitter production, which have been implicated in the onset and severity of depressive symptoms (Foster et al., 2021). A growing body of research, including findings by Kumar et al. (2023) has identified specific alterations in microbial genera associated with depression. Consistent reductions have been observed in beneficial taxa such as Clostridia, *Bacteroides*, *Alistipes*, *Roseburia*, *Coprococcus*, *Dialister*, *Faecalibacterium*, and *Butyricicoccus*. These bacteria are often involved in the production of anti-inflammatory SCFAs and the regulation of neurotransmitter pathways. Conversely, increased levels of potentially pathogenic or pro-inflammatory genera such as *Proteobacteria*, *Actinobacteria*, *Clostridium*, *Streptococcus*, and *Oscillibacte*r have been noted in individuals with depression. Among these findings, Firmicutes and Actinobacteria appear to exhibit consistent patterns, with a notable decline in Firmicutes (particularly SCFA-producing species like *Faecalibacterium*) and a proliferation of certain Actinobacteria (e.g., *Eggerthella*) in depressed populations. Interestingly, some genera like *Bacteroides* and *Clostridium* show variability, with their abundance fluctuating based on factors such as individual genetics, diet, and environmental exposures, as well as study design and population demographics. Table 1 summarized the classification of the bacteria genera towards human and animal studies.

**TABLE 1:** A summary of the bacterial genera and their roles in short chain fatty acid (SCFA) levels and classification towards human and animal studies.

Bacteria genera	Human studies	Animal studies	Remarks
*Firmicutes*	Research suggests that the make-up of gut microbiota is different in people whoa are diagnosed with depression (Valles-Colomer et al., 2019)	In animal models of depressive states, researcher found loss of *Firmicutes* and changes to microbiota, suggesting that dysbiosis can lead to depressive-live behaviours (Zheng et al., 2016)	Key SCFA producers (e.g., *Faecalibacterium*, *Coprococcus*) (Valles-Colomer et al., 2019, Zheng et al., 2016)
*Bifidobacterium*	Indicates that *Bifidobacterium* levels are significantly reduced in depressed individuals as compared with healthy controls (Huang and Liu, 2024)	In faecal microbiota, abundance of *Bifidobacterium* was substantially reduced in the cases exhibiting depression-like behaviour compared to healthier cases (Sharma et al., 2021)	The analysis indicates that *bifidobacteria* may increase serotonin in the gastrointestinal tract, possibly influencing central nervous system function, as an enhancement in mood (Huang and Liu, 2024, Sharma et al., 2021)
*Bacteroides*	Decreased in some cases, however it also increased in others. Nguyen et al. found a consistent drop in levels of *Bacteroides* observed in patients with chronic schizophrenia which can be comorbid with depressive disorders (Nguyen et al., 2019)	Guilherme et al. found that in wild-type mice experiencing social stress, changes in *Bacteroides* populations were associated with resistance to depressive symptoms, suggesting the possibility the genus can alter neurochemical pathways (Guilherme et al., 2022)	Variables. Decreased populations of these bacteria have been associated with more depressive symptoms in some studies while other studies suggest that specific *Bacteroides* species may have protective effects against mood disorders (Mottawea et al., 2020)
*Clostridium*	*Clostridium* has been tied to symptom severity in affective disorders and anxiety disorders, providing further evidence of the significance of gut microbiota composition in psychopathology (Scala et al., 2025)	Some studies suggest that chronic stress can change gut microbiome composition significantly, including levels of *Clostridium* which could be influential in immunomodulation in the context of mood disorders (Park et al., 2023)	Participates in SCFAs production (Cheng et al., 2024)
*Faecalibacterium*	There was a considerable reduction in the concentration of *Faecalibacterium* in patients diagnosed with MDD in similar samples to healthy controls (Angelova et al., 2023)	Indicated the germ-free mice inoculated in which ‘depression microbiota’ had been associated, were missing the beneficial *Faecalibacterium* and likely had other detrimental species (Zheng et al., 2016)	*Faecalibacterium prausnitzii* is considered a large donor of butyrate in the human gut microbiome (Yang et al., 2023)
*Coprococcus*	Individuals who had a lower abundance of *Coprococcus* were significantly more likely to have higher levels of depressive symptoms (Valles-Colomer et al., 2019)	A microbial intervention focusing on *Coprococcus* strains given to chronic stress subject mice produced alterations in anxiety-like behaviour and depressive-like symptoms (Valles-Colomer et al., 2019)	*Coprococcus*, produces neuroactive compounds like 3,4-dihydroxyphenylacetic acid, that have been associated with dopamine modulation, a well-known neurotransmitter involved in mood regulation (Valles-Colomer et al., 2019)
*Roseburia*	Lee et al.’s pilot study tracked changes in gut microbiota in elderly patients receiving antidepressant treatment, it showed that changed in variants such as *Roseburia* can predict treatment outcome. Hence, it’s possible to propose that changes in gut microbiota can predict psychiatric treatment outcomes (Lee et al., 2022)	There is research that supports the administration of probiotic including those containing *Roseburia* provide improvement in behavioural outcomes for stress and anxiety (Cheung et al., 2019)	*Roseburia* also has anti-inflammatory properties that may attenuate neuroinflammatory processes related to depressive disorder (Yao et al., 2022)
*Dialister*	A lower abundance of *Dialister* observed in the fecal microbiota of patients with MDD, in comparison to healthy controls, indicates that *Dialister* may have utility as a self-reportable biomarker for depression (Zheng et al., 2016)	Use of probiotics containing *Dialister* are associated with a reversal of depressive-like behaviours, and which seemingly return levels of neuroinflammatory markers, typically correlated with mood disturbances which is back to baseline (Zheng et al., 2016)	Low fibre diets are associated with lower SCFA production, which is linked to loss of beneficial bacteria including *Dialister*, an essential species to maintain mental health (Al-Amrah et al., 2023)
*Lactobacillus*	Dawe et al. found that pregnant women in randomized controlled trial who received *Lactobacillus rhamnosus* HN001 had a much lower postpartum anxiety and depression score than the placebo group (Dawe et al., 2020)	The oral administration of *Lactobacillus casei* not only improved behavioural characteristics associated with a depressed state, but it also restored beneficial levels of brain-derived neurotropic factor (BDNF) and restored BDNF pathways to promote neuroplasticity and appropriate mood (Choi et al., 2022)	*Lactobacillus* also upregulates the biosynthesis of gamma-aminobutyric acid that has a potential neuroprotective action that might have a physiological mechanism in stabilizing mood (Dong et al., 2022)
*Ruminococcus*	Individuals with MDD showed a severe depletion in the *Ruminococcus* genus, with promising potential as a depression biomarker (Maes et al., 2022)	A negative correlation of *Ruminococcus* with depressive states in a rodent model (Maes et al., 2022)	*Ruminococcus* species have been directly associated with dopaminergic metabolism indicating potential influence on mood regulation (Yang et al., 2021)
*Alistipes*	Patients struggling with MDD had decreased levels of *Alistipes* when compared to healthy controls (Li et al., 2023; Zhang et al., 2022; Liu et al., 2022a)	Fluctuations in the metabolism of *Alistipes* were associated to depressive-like behaviours in animal models (Li et al., 2023)	Alistipes has the ability to hydrolyze tryptophan to indole, which may reduce the availability of serotonin, a neurotransmitter involved neurological processes associated with mood (Prochazková et al., 2021)
*Prevotella*	Clinical observations have indicated that patients suffering from MDD had lower levels of *Prevotella* in comparison to a healthy control population (Zhu et al., 2021a; McGuinness et al., 2022)	One study observed that fluoxetine treatment during pregnancy and lactation, which is a common antidepressant, resulted in greater abundance of *Prevotella* in a rat model with depressive-like behaviour (Ramsteijn et al., 2020)	*Prevotella* species are associated with plant-rich diets that are high in carbohydrates and fibre (Luo et al., 2022)

Collectively, these data suggest that microbial signatures associated with MDD are not only diverse but also context-dependent, highlighting the importance of personalized approaches when examining microbiota-based interventions for mental health. Additionally, research has shown that exposure to specific microbial strains can protect against stress-induced behavioral changes and systemic immune alterations, offering compelling evidence for the potential of microbe-based therapies in treating stress-related disorders (Nakhal et al., 2024).

SCFAs, including acetate, propionate, and butyrate, are metabolites produced by a healthy gut microbiota during the fermentation of dietary fibers and resistant starch (Silva et al., 2020). These SCFAs play a crucial role in immune modulation (Cheng et al., 2024). Butyrate, in particular, is thought to influence the gut-brain axis, potentially by enhancing colonic serotonin production, a key neurotransmitter involved in mood and behavior regulation. Additionally, animal studies suggest that butyrate may exert antidepressant-like effects by stimulating the production of brain-derived neurotrophic factor (BDNF), a protein essential for neuronal development and survival (Mansuy-Aubert and Ravussin, 2023).

Moreover, SCFAs are vital for reducing inflammation, maintaining intestinal barrier integrity, and regulating central nervous system function. However, microbial imbalance, or dysbiosis, can reduce SCFA production, diminishing their anti-inflammatory effects and promoting inflammation. Research indicates that restoring SCFA levels through dietary interventions or probiotic supplementation can enhance immune function and alleviate depressive symptoms. These findings highlight the therapeutic potential of modulating gut microbiota for treating depression (Mansuy-Aubert and Ravussin, 2023).

Given the urgent need for novel treatments and preventative strategies for mental health (Viana, 2024). Understanding the influence of the gut microbiota on emotional and cognitive functions, neurotransmitter synthesis, neuroinflammation, and gut barrier integrity is critical (Singh et al., 2024). This complex relationship involves bidirectional communication between the gut and the brain, with brain regions such as the insula and anterior cingulate cortex playing key roles in regulating both gut function and psychological responses (Kano and Fukudo, 2025). Despite variations in gut microbiota composition across studies, all evidence consistently demonstrates significant alterations in the gut microbiota of individuals with depression, suggesting that the gut microbiota may be a promising target for both the prevention and treatment of this disorder (Xiong et al., 2023). A comprehensive understanding of the gut microbiota’s influence on psychiatric conditions is vital for developing innovative and effective therapeutic strategies, emphasizing the need for an integrated approach to mental healthcare (Singh et al., 2024).

The gut microbiota is integral to the synthesis and regulation of neurotransmitters such as serotonin, dopamine, and glutamate, which are critical for neurological and immunological functions in the brain. This complex microbial community is diverse, with certain species potentially promoting mental wellbeing, while others may contribute to the development and progression of mental disorders (Xiong et al., 2023). Studies have revealed significant alterations in the gut microbiota composition of individuals with depression compared to healthy controls. Depressed patients showed reduced levels of *Dialister* and *Coprococcus* species, alongside elevated levels of *Prevotella*, *Klebsiella*, *Streptococcus*, and *Clostridium XI*, as well as decreased levels of Bacteroidetes. Animal studies supported these findings, showing that fecal microbiota transplants from depressed individuals induced depression-like behaviors in mice, while transplants from healthy rats prevented depression in susceptible rats. These results suggest that gut microbiota dysbiosis plays a significant role in depression by influencing protein expression along the gut–brain axis. While microbial composition varies across studies, certain families (e.g., *Paraprevotella*-positive and *Streptococcaceae* and *Gemella*-negative) and genera (e.g., *Prevotella*, *Klebsiella*, and *Clostridium*) have been consistently associated with depression (Xiong et al., 2023). Emerging evidence suggests that gut microbiome dysbiosis plays a crucial role in the neurovascular pathophysiology of glymphatic system dysfunction and cerebral small vessel disease, which may exacerbate neuroinflammation and impair the BBB function, both of which are critical processes in the development and progression of depression (Che Mohd Nassir et al., 2024).

## 3 Current evidence linking the gut microbiota to depression

### 3.1 Microbiota composition and diversity

Microbial diversity serves as a key indicator of gut ecosystem health, which is typically measured as alpha diversity (the richness and evenness of a sample) and the beta diversity (variance across groups). Less microbial diversity has been linked to many disease states, including MDD, in which patients frequently have less alpha diversity than healthy controls (Shen et al., 2021; Liu et al., 2023). This brings up the question of how much of the microbial change is due to antidepressant medication as opposed to the disease itself. The human gastrointestinal tract harbors a complex ecosystem of billions of microorganisms, collectively referred to as the gut microbiota, predominantly composed of bacteria. This microbial community plays a vital role in various physiological processes, including maintaining intestinal barrier integrity, regulating energy metabolism, defending against pathogens, and modulating immune function (Noh et al., 2021). While overall diversity and phylum-level analyses may not consistently reveal significant differences, studies comparing the gut microbiota of individuals with depression to healthy controls have identified specific bacterial taxa associated with this mental health condition (Gao et al., 2023).

MDD is often linked to a decrease in microbial diversity, characterized by lower levels of beneficial bacteria such as *Bacteroides* and *Faecalibacterium* in individuals with depression. Fluctuations in the relative abundance of *Bacteroides* have been observed in depressed individuals, with medication-free individuals exhibiting higher levels, suggesting a potential relationship between this bacterium and depressive symptoms. *Faecalibacterium*, a key producer of the anti-inflammatory SCFA butyrate, is significantly reduced in individuals with depression. This decline in beneficial microorganisms may contribute to the inflammatory processes observed in depression (Gao et al., 2023). Consistent findings across studies have revealed lower abundances of several beneficial bacterial genera, including *Butyricicoccus*, *Coprococcus*, *Faecalibacterium*, *Fusicatenibacter*, *Eubacterium ventriosum* group, *Romboutsia*, and *Subdoligranulum* in individuals with depression compared to healthy controls. Conversely, higher abundances of *Eggerthella*, *Enterococcus*, *Escherichia*, *Flavonifractor*, *Holdemania*, *Lachnoclostridium*, *Paraprevotella*, *Rothia*, and *Streptococcus* have been observed in individuals with depression. Several hypotheses have been proposed to explain these associations, primarily focusing on altered pro-inflammatory and anti-inflammatory signaling along the gut-brain axis (Gao et al., 2023).

#### 3.1.1 Preclinical evidence

Research in animal models indicates that gut microbiota has a profound effect on stress responses, anxiety-like behaviors, depressive behaviors, and social behaviors. Neurological immunological, and endocrine pathways are mechanisms that could support the gut-brain link. Microbes can produce neuroactive compounds and affect host neurotransmitter signaling. Zhou et al. (2023) indicated that chronic unpredictable mild stress in animal models produces changes in depressive behaviours as well as composition of gut microbiota can regulate the physiological features of depression (Zhou et al., 2023). Similarly, studies involving animals have demonstrated that changes in gut microbiome can affect the action of antidepressants, and researchers have shown that certain antidepressants influence both the microbiome and the metabolism related to depression (Chevalier et al., 2020; Zhang W. et al., 2021).

#### 3.1.2 Clinical evidence

In a clinical study of 90 young people in the United States, investigators compared gut microbiomes between 43 patients with MDD and healthy 47 controls. There were significant differences in gut microbiota community composition between groups across several taxonomic levels. On a phylum level, MDD patients showed lower levels of *Firmicutes* and higher levels of *Bacteroidetes*, with similar trends found in the class which are *Clostridia* and *Bacteroidia* and order levels as well (Liu et al., 2020). Ling et al. conducted a case-control study and determined participants who had experienced depression and whose faecal microbiota were significantly different than healthy control groups, confirming the notion that depressives’ symptoms are associated with specific gut microbiota profiles (Ling et al., 2022). The literature suggests there is often a correlation between depressive disorders and diminishing microbial diversity. Liu L. et al. (2022) also detected a negative association between microbial alpha-diversity and the severity of the depression symptoms measured in their study, suggesting people with more variety in their gut bacteria reported lower depressive symptoms (Liu L. et al., 2022). Also, Zhu J. et al. (2021) determined patients with anxiety and depression, had substantially less alpha diversity than healthy controls, demonstrating the link between diminished gut microbiota diversity and mental health disorders (Zhu J. et al., 2021).

### 3.2 Prebiotics, synbiotics, postbiotics, and probiotics

A review examined the role of probiotics, prebiotics, and postbiotics in alleviating depressive symptoms through modulation of the GBA (Luqman et al., 2024). In adults, the gut microbiota, primarily residing in the colon, can weigh up to approximately 1 kg. The dominant phyla within this complex ecosystem include *Bacteroidetes* and *Firmicutes*, while *Actinobacteria*, *Proteobacteria*, and *Verrucomicrobi*a are present in smaller proportions. Additionally, the gut microbiome includes methanogenic archaea, eukaryotes (mainly yeast), and numerous bacteriophages (Bonaz et al., 2018). Probiotic consumption can help restore the balance of the gut microbiota (Alli et al., 2022). These beneficial microorganisms can positively influence the immune system, which is crucial for mood regulation. Depression and anxiety are often associated with dysregulated immune responses, characterized by increased inflammation. Probiotics may help mitigate this imbalance by stimulating anti-inflammatory responses and reducing the production of pro-inflammatory molecules (Rahmannia et al., 2024).

Prebiotics are non-digestible compounds, such as fructooligosaccharides, galacto-oligosaccharides, and xylooligosaccharides, that serve as food for gut microbes, thereby altering the gut microbiome composition in a manner that benefits the host (Bakshi et al., 2024; Bindels et al., 2015). They selectively stimulate the growth and/or activity of specific beneficial bacteria within the intestine, promoting improved host health (Bindels et al., 2015; Bevilacqua et al., 2024). Several studies have demonstrated the potential of prebiotics to influence stress, anxiety, and depression, possibly through a reduction in perceived stress associated with changes in *Bifidobacterium* spp. or other gut microbiota taxa (Bevilacqua et al., 2024).

Synbiotics synergistically combine probiotics and prebiotics (Alli et al., 2022). The primary objective of synbiotic supplementation is to enhance gut health by introducing beneficial bacteria (probiotics) and providing essential nutrients (prebiotics) to support their survival, growth, and activity within the digestive system. Meanwhile, postbiotics refer to substances formed as a result of formerly living microorganisms or inactivated microorganisms. A postbiotic may be composed of non-living, whole cells or structural elements of bacteria (e.g., cell walls). A postbiotic must be derived from a well-defined organism or mixture of organisms, with known genomic sequences, and produced using a reproducible technical approach to biomass production and inactivation (Vinderola et al., 2022). While probiotics are living microorganisms, postbiotics are composed of non-living microbial cells or their components, such as SCFAs, peptides, and polysaccharides. These substances can influence brain function and mood modulation through several potential mechanisms. Recent evidence suggests that some postbiotics may be superior and safer alternatives to probiotics for alleviating depressive symptoms. While research on postbiotics-based interventions for depression is still in its early stages, the evidence supporting their anti-depressive potential is promising (Liu et al., 2023).

Postbiotics can act on the immune system to reduce pro-inflammatory cytokines and increase anti-inflammatory responses. The reduction of systemic inflammation is crucial because chronic inflammation has been linked to the pathophysiology of depression (Chudzik et al., 2021). Postbiotics, such as SCFAs, have been shown to enhance the expression of tight junction proteins, improving gut barrier integrity and reducing intestinal permeability (Zhang et al., 2025). Additionally, postbiotics help preserve gut barrier function, preventing the translocation of lipopolysaccharides (LPS) into circulation, where they can lead to systemic inflammation and depressive symptoms (Varesi et al., 2023).

**FIGURE 1:**
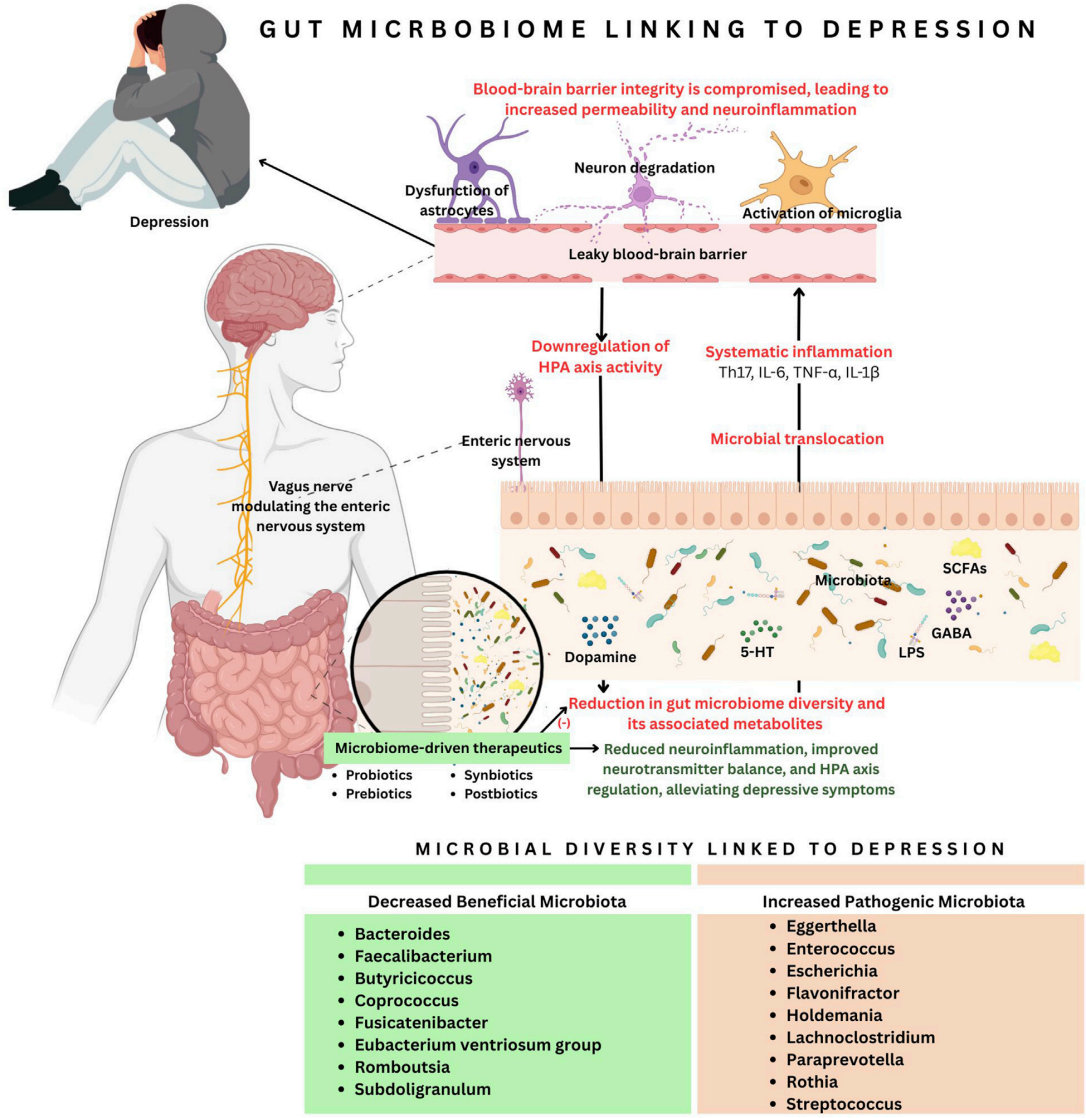
The connection between gut microbiota and depression highlights how microbial imbalance contributes to neuroinflammation and neurotransmitter dysregulation. A reduction in beneficial microbiota and an increase in pathogenic bacteria lead to microbial translocation, systemic inflammation (Th17, IL-6, TNF-α, IL-1β), and a leaky blood-brain barrier (BBB). This triggers neuron degeneration, microglial activation, and astrocyte dysfunction, resulting in reduced dopamine, serotonin, and gamma-aminobutyric acid (GABA) levels. Additionally, gut dysbiosis downregulates the hypothalamic-pituitary axis (HPA) and disrupts vagus nerve stimulation (VNS). Microbiota-driven therapeutics, such as probiotics, prebiotics, synbiotics and postbiotics, are a potential intervention to restore microbial imbalance and alleviate depressive symptoms.

Probiotic compositions typically include diverse strains of *Lactobacillus* and *Bifidobacterium*, which have been shown to reduce inflammation and modulate immune responses by inhibiting the production of the cytokine IL-8 in human colon epithelial cells and decreasing intestinal permeability, thereby preventing endotoxemia (Alli et al., 2022). Prebiotics, such as dietary fiber, promote the growth of Lactobacilli and Bifidobacteria (Carlson et al., 2018). Animal studies have shown that supplementation with *Bifidobacterium infantis* can alleviate stress and depression in rats, while a 28-day course of *Lactobacillus rhamnosus* supplementation has been shown to reduce depressive symptoms in humans (Alli et al., 2022). Diets high in glucose, fructose, and sucrose can significantly alter the gut microbiota composition, marked by a dramatic increase in *Bifidobacterium* and a substantial decrease in *Bacteroides*. Studies in mice fed a fructose-rich diet have shown significant increases in *Coprococcus*, *Ruminococcus*, and *Clostridium*, while simultaneously observing a reduction in the Clostridiaceae family (Cryan et al., 2019). Figure 1 demonstrates the connection between gut microbiota and depression, highlighting how microbial imbalance contributes to neuroinflammation and neurotransmitter dysregulation. Also, to enhance clarity and evidence synthesis, support claims are presented in Table 2.

**TABLE 2:** Expanded support claims evidence: Prebiotics strains, Synbiotics strains, Postbiotics strains, Probiotics strains and Diet.

Intervention	Strain/Example	Claim	Supporting evidence	References
Prebiotics	• Fructooligosaccharides (FOS) • Galactooligosaccharides (GOS)	Enhance *Bifidobacterium* growth and reduce stress	Linked to reduced perceived stress and improved gut composition	Bakshi et al. (2024), Bindels et al. (2015), Bevilacqua et al. (2024)
	Dietary fiber	Promotes *Lactobacilli* and *Bifidobacteria* growth	Improves microbiota composition and supports mood regulation	Carlson et al. (2018)
Synbiotics	Combination of probiotics and prebiotics	Synergistically improve gut health and mood	Probiotics colonize more effectively with prebiotic support	Alli et al. (2022)
Postbiotics	SCFAs (e.g., butyrate)	Strengthening gut barrier and reduce systemic inflammation	Increase tight junction protein expression, reduce intestinal permeability	Zhang et al. (2025), Varesi et al. (2023)
	Peptides, polysaccharides	Modulate immune response and reduce pro-inflammatory cytokines	Reduce systemic inflammation linked to depression	Liu et al. (2023), Vinderola et al. (2022), Chudzik et al. (2021)
Probiotics	*Lactobacillus rhamnosus*	Reduces depressive symptoms in humans after 28-day supplementation	Shown to reduce depressive symptoms in clinical trial	Alli et al. (2022)
	*Bifidobacterium infantis*	Alleviates stress and depression in animal models	Supplementation improved behavioral outcomes in rats	Alli et al. (2022)
	Mixed strains (*Lactobacillus* & *Bifidobacterium*)	Modulate immune responses and reduce inflammation	Inhibit cytokine IL-8 in colon epithelial cells, decrease intestinal permeability, prevent endotoxemia	Alli et al. (2022), Rahmannia et al. (2024)
Dietary Influence	High sugar diet (glucose, fructose, sucrose)	Induces dysbiosis and increases depression risk	Increases *Bifidobacterium*, *Coprococcus*, *Ruminococcus*, *Clostridium*; decreases *Bacteroides*, *Clostridiaceae*	Cryan et al. (2019)

## 4 How depression alters the gut microbiota

Depression alters the composition and activity of the gut microbiota through several interconnected pathways, particularly inflammation, neurotransmitter modulation, and stress responses. Depressive states promote pro-inflammatory conditions that favour gut microorganisms associated with inflammation while inhibiting species that exert anti-inflammatory effects (Liu et al., 2024). Research on human depression has shown that depressive moods are linked to an increase in *Bacteroides* (pro-inflammatory) and a decrease in *Clostridium* species (SCFA producers), which affects butyrate metabolism (Liu et al., 2023). A lack of butyrate contributes to intestinal barrier dysfunction, leading to endotoxemia and systemic inflammation, which are further exacerbated by the depressive state (Liu et al., 2024). Additionally, there is an increase in *Eggerthella* and *Ruminococcaceae* species, which are associated with impaired glutamate metabolism, and a decrease in *Coprococcus* and *Dialister* species, which are involved in dopamine production (Radjabzadeh et al., 2022).

Furthermore, stress hormone signalling plays a crucial role in gut microbiota alterations. Microbial changes may occur early in MDD and could be part of its onset. Over time, pathogenic shifts in the gut microbiota contribute to dysbiosis by altering the gut environment (Liu et al., 2023). The activation of the HPA axis in depression increases cortisol production, which in turn affects gastrointestinal motility and secretion (Averina et al., 2024). These stress hormones also alter the gut environment, favouring certain pathogenic bacteria while disrupting beneficial SCFA-producing species (Bevilacqua et al., 2024; Madison and Kiecolt-Glaser, 2019). As a result, intestinal permeability (also known as “leaky gut”) increases, facilitating bacterial translocation (Kumar et al., 2023; Madison and Kiecolt-Glaser, 2019).

Dysbiosis in depression is also linked to neurotransmitter disturbances. Specific microbiota associated with depression shows reductions in neurotransmitter synthesis. For example, the depletion of *Coprococcus* results in a diminished supply of dopamine precursors, and strains like *Coprococcus comes* and *Coprococcus catus* are involved in the synthesis of DOPAC, a dopamine metabolite (Hamamah et al., 2022). Additionally, reductions in *Subdoligranulum* have been correlated with disruptions in GABA production (Radjabzadeh et al., 2022). Likewise, Bellach et al. (2024) noted that depletion of *Subdoligranulum* and other genera, such as *Coprococcus* and *Faecalibacterium,* may potentially serve as transdiagnostic markers of psychopathologies (Bellach et al., 2024). Also, Park et al. (2023) found that dysbiosis with *Subdoligranulum* associated with depressive symptoms, providing further support for these findings. They suggested that certain bacterial taxa, including *Subdoligranulum*, participate in neurotransmitter biosynthesis including GABA synthesis (Park et al., 2023). This implies that the presence of these gut microbes is significant for emotional regulation. Inhibited tryptophan metabolism also negatively affects serotonin production. Serotonin (5-HT) has been implicated in the development and treatment of depression, and its levels are regulated by the gut microbiota (Irum et al., 2023).

Alterations in microbial metabolites further contribute to depression-related changes in the gut. Depression has been shown to alter the gut metabolome by reducing SCFA synthesis, with 40%–60% less butyrate present in MDD patients (Liu et al., 2023; Liu et al., 2024). Additionally, the accumulation of neurotoxic metabolites such as p-cresol sulphate contributes to gut microbiota alterations in depression. *p-Cresol* has been shown to inactivate dopamine beta-hydroxylase, an enzyme necessary for converting dopamine into norepinephrine. This disruption can lead to neurotransmitter imbalances, which affect mood regulation and cognitive functions (Flynn et al., 2025).

Further research indicates that depressed individuals often exhibit an overabundance of bacteria responsible for producing phosphatidylcholines, contributing to gut dysbiosis and systemic toxicity that impair normal brain functioning (Xie et al., 2024). Disrupted bile acid metabolism has also been associated with depression. Secondary bile acids, modulated by the gut microbiota, are negatively correlated with the severity of depressive symptoms, suggesting that higher levels of bile acids may have a protective effect against depression (Liu et al., 2023).

**FIGURE 2:**
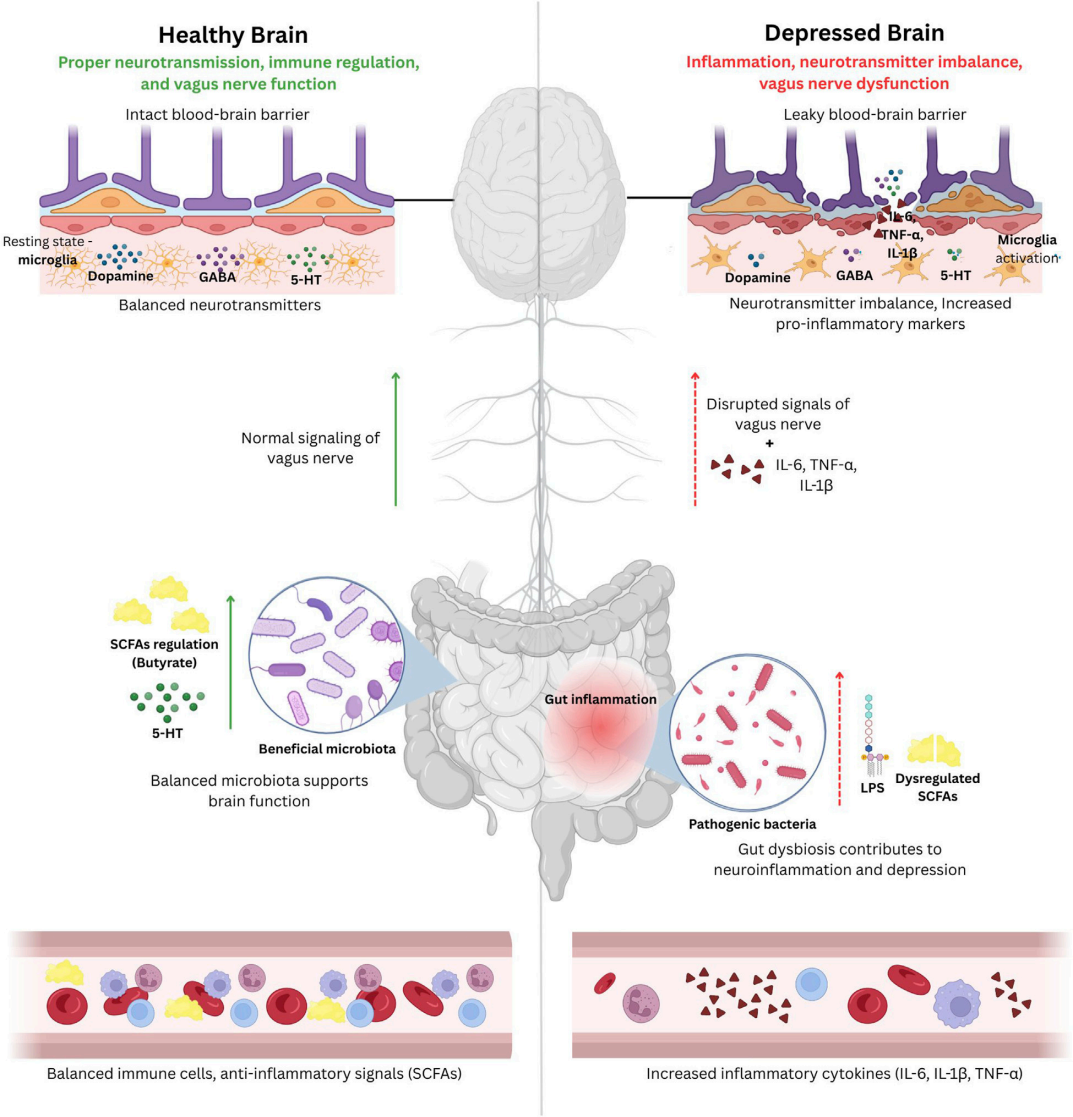
A schematic illustration of the difference between a healthy brain and a depressed brain and emphasizing the role of the gut-brain axis (GBA). The healthy brain (on the left) can maintain proper neurotransmission, regulation of the immune system, and vagus nerve regulation, all of which is supported by an intact blood-brain barrier, proper levels of neurotransmitters (dopamine, gamma-aminobutyric acid (GABA), serotonin) and ant-inflammatory signals (SCFAs produced by gut bacteria), and beneficial gut bacteria. Whereas, the depressed brain (on the right), will exhibit neuroinflammation, neurotransmitter imbalance, and vagus nerve dysfunction due to leaky blood-brain barrier, activation of microglia, increased levels of pro-inflammatory cytokines such as interleukins (IL-6, IL-1β) and tumor necrosis factor alpha (TNF-α), and gut dysbiosis leading to systemic inflammation. This demonstrates the impact of gut health on depression.

Studies on depression treatments also demonstrate the influence of gut microbiota. For example, FMT from depressed individuals to germ-free rats successfully transmits depression traits, providing evidence for a causal relationship between the gut microbiota and depression (Irum et al., 2023). Conversely, supplementation with *Clostridium butyricum*, a butyrate-producing bacterium, has shown antidepressant properties by restoring gut microbial balance and reducing inflammation (Liu et al., 2024). These alterations in the gut microbiota create a self-perpetuating cycle, in which the conditions of depression worsen gut dysbiosis, neuroinflammation, and neurotransmitter imbalances. Figure 2 illustrates the difference between a healthy brain and a depressed brain and emphasizes the role of the GBA.

## 5 Mechanism of action

### 5.1 Neuroimmune modulation

Neuroinflammation, a hallmark of many neurological disorders, involves the activation of microglia and astrocytes in response to infections, trauma, and protein aggregation (e.g., amyloid-beta) (Zhang W. et al., 2023). These glial cells release pro-inflammatory cytokines and reactive oxygen species (ROS), contributing to neuronal damage. Conversely, neuroimmune regulation mediated by interactions between the nervous and immune systems helps maintain cytokine balance, protect the BBB, and limit microglial overactivation (Hein et al., 2020).

The GBA plays a central role in this process, with gut microbes producing SCFAs that modulate immune cells and neuroinflammation (Bostick et al., 2022). Gut dysbiosis disrupts this balance via increased lipopolysaccharide (LPS) translocation, triggering systemic inflammation and potentially altering blood-brain barrier permeability to psychoactive drugs (Clapp et al., 2017). This immune signaling, often driven by cytokines, can cross the BBB and promote neuroinflammation, a key factor in depression and other neuropsychiatric conditions (Medina-Rodriguez and Beurel, 2022).

Circadian rhythm disruption and chronic stress further exacerbate gut dysbiosis and neuroimmune dysfunction, impairing neurogenesis and increasing vulnerability to mood disorders (Ullah et al., 2024). Inflammatory depression, marked by elevated cytokines and altered gut microbiota (e.g., increased *Bacteroides*, decreased *Clostridium*), exemplifies this gut-immune-brain interaction (Liu et al., 2024; Zhang K. et al., 2023).

### 5.2 Neurotransmitter production

Neurotransmitters such as serotonin, dopamine, GABA, and glutamate are critical for brain function and are heavily influenced by gut microbiota. Although most serotonin (∼90%) is produced peripherally by enterochromaffin cells in the gut, its precursor tryptophan can cross the BBB for central synthesis (Chen et al., 2021). Gut microbes modulate tryptophan metabolism and influence neurotransmitter availability through several mechanisms, including direct precursor production, enzymatic catalysis, and signaling via microbial metabolites (Miri et al., 2023).

For example, spore-forming bacteria can enhance serotonin production by up-regulating TPH1 in enterochromaffin cells (Chen et al., 2021). Inflammatory pathways, particularly the kynurenine pathway activated by cytokines like IL-6, divert tryptophan from serotonin synthesis toward neurotoxic metabolites contributing to depression. Several bacteria (e.g., *Streptococcus*, *Lactobacillus*, *Eubacterium coli*) can also synthesize serotonin directly (Reyes-Martínez et al., 2023).

### 5.3 Vagus nerve pathways

The vagus nerve, the main conduit of gut-brain communication, is composed primarily of afferent fibers that relay sensory signals from the gut to the brain (Cao et al., 2024). These signals are processed in the nucleus tractus solitarii and influence emotion, behavior, and physiological homeostasis (Berthoud et al., 2021). Efferent vagal fibers transmit brain signals to the gut, regulating motility, secretion, inflammation, and even microbiota composition (Yu et al., 2020).

**FIGURE 3:**
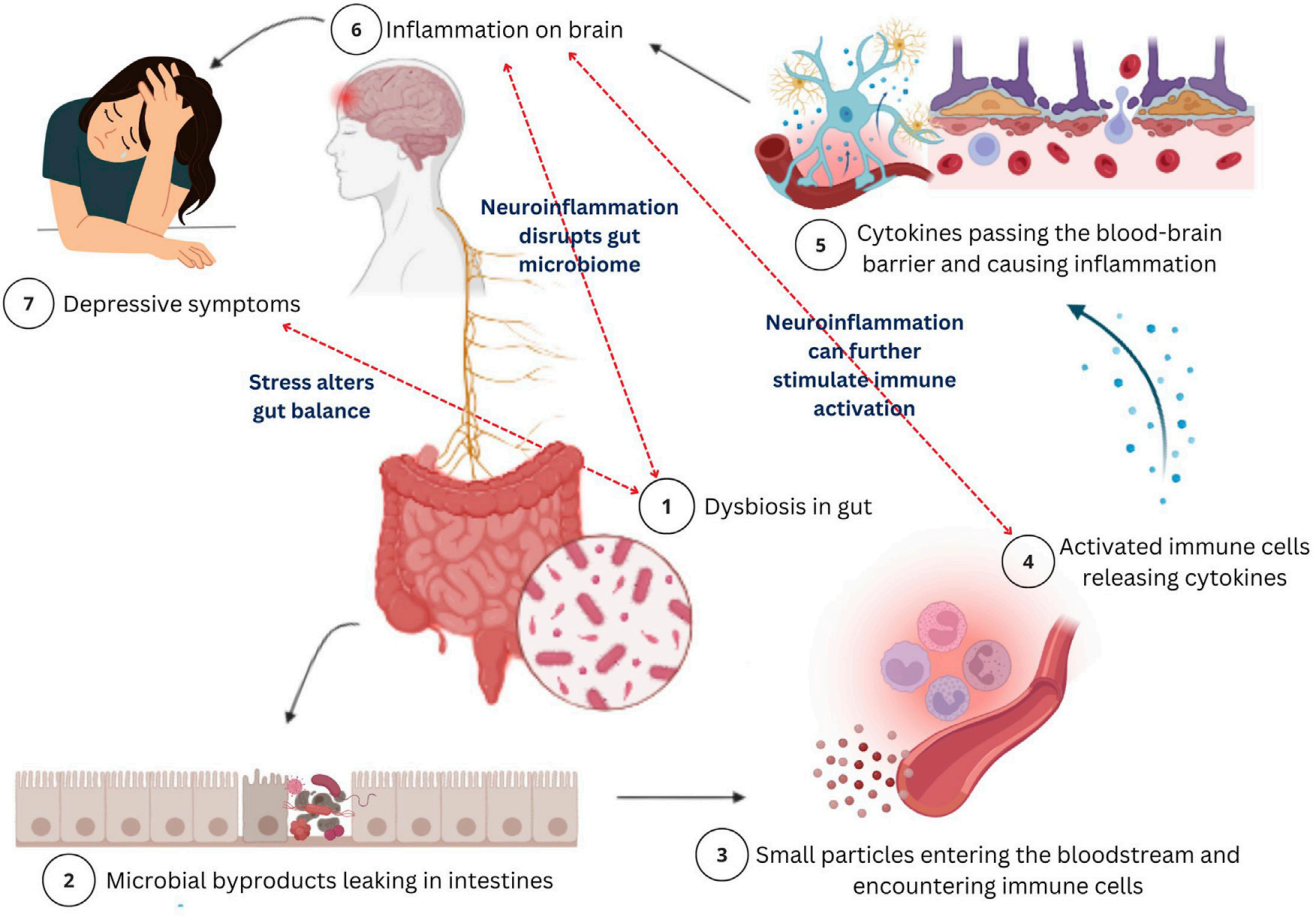
A schematic illustration of the mechanism of action of the gut-brain axis and its role in depression. Gut dysbiosis, an imbalance in gut bacteria (1) disrupts the normal gut function which leads to microbial byproducts leaking in intestines (2) and entering the bloodstream, allowing harmful particles to enter the bloodstream (3). These substances trigger an immune response, causing the release of cytokines (4). Further, these cytokines will travel to the brain by crossing the blood-brain barrier (BBB) (5) and causing inflammation (neuroinflammation) (6). This brain inflammation disrupts neurotransmitters and damages neurons, potentially leading to depression. The bidirectional nature is highlighted as neuroinflammation disrupts gut microbiota, exacerbating dysbiosis, while stress further alters gut balance, perpetuating this cycle of immune activation, inflammation, and depression. This process illustrates the connection between gut health and mental health, showing how gut bacteria imbalances can contribute to mood disorders through immune system dysfunction.

Gut-derived metabolites can stimulate vagal afferents, allowing microbial signals to influence brain activity (Jameson et al., 2024). This bidirectional feedback loop is integral to maintaining gut-brain homeostasis. Disruption through stress or dysbiosis can suppress vagal activity and activate the sympathetic nervous system, contributing to gastrointestinal and psychiatric disorders (Bonaz et al., 2018). Vagus nerve stimulation (VNS) has shown promise in treatment-resistant depression (Nahas et al., 2007). Clinical studies and neuroimaging suggest VNS modulates activity in mood-regulating brain regions such as the ventromedial prefrontal cortex, supporting its role in restoring emotional balance (Breit et al., 2018). Figure 3 illustrates the mechanism of action of GBA and its role in depression.

## 6 Specific microbial markers in depression

Mounting evidence has highlighted gut microbiota’s role in influencing neurochemical pathways involved in depression specifically through the modulation of neurotransmitters such as butyrate, GABA, glutamate, and serotonin (Radjabzadeh et al., 2022). Preclinical and clinical studies increasingly support the idea that microbial dysbiosis may contribute to the onset and progression of depression by disrupting these pathways (Radjabzadeh et al., 2022).

Recent research has identified specific microbial taxa associated with depressive symptoms, underscoring the intricate relationship between gut microbiota and mental health. A consistent finding across studies is the reduced abundance of butyrate-producing bacteria in individuals with MDD (Radjabzadeh et al., 2022; Modesto Lowe et al., 2023; Zhu L.-B. et al., 2021). Butyrate, a SCFA, plays a key role in maintaining intestinal barrier integrity, modulating inflammation, and supporting neurogenesis. It is commonly produced by Gram-positive anaerobic bacteria in the human colon through the fermentation of dietary fibers (Louis and Flint, 2009).

Butyrate can modulate the GBA via epigenetic mechanisms, such as upregulating cholinergic neuron expression, and can influence brain function by crossing the BBB and activating the vagus nerve and hypothalamus (Radjabzadeh et al., 2022). Several bacterial genera, including *Faecalibacterium*, *Butyricimonas*, *Coprococcus*, and *Dialister*, have been identified as key butyrate producers. Their depletion has been consistently associated with depressive symptoms (Singh et al., 2024; Radjabzadeh et al., 2022; Modesto Lowe et al., 2023). In particular:

1. *Eggerthella* and *Actinomyces* are increased in depression and correlate with lower levels of butyrate-producing bacteria (Suda and Matsuda, 2022).
2. Enterobacteriaceae, opportunistic pathogens enriched in depression, are often found alongside depleted levels of beneficial taxa such as *Faecalibacterium* (Gao et al., 2023).
3. Butyrate-producing microbes help maintain an anaerobic gut environment, which suppresses pathogenic colonization (e.g., *Salmonella*, *E. coli*) by increasing epithelial oxygen demand and preventing aerobic pathogen growth (Viana, 2024).

A large population-based study (Flemish Gut Flora Project, *n = 1,054*) and other investigations have shown that individuals with depression often exhibit reduced levels of *Coprococcus* and *Dialister*, alongside increased pro-inflammatory species such as *Desulfovibrio* (Modesto Lowe et al., 2023; Matsuzaki et al., 2024).

### 6.1 GABA-producing microbes

GABA is the brain’s primary inhibitory neurotransmitter and plays a critical role in mood regulation by counterbalancing excitatory signals like glutamate. Disruptions in GABA signaling are implicated in anxiety and depression. Certain gut bacteria can produce GABA, and their presence or absence may influence depressive symptoms via the gut-brain axis. For example:

Preclinical Evidence:

1. *Lactobacillus rhamnosus* has been shown to modulate GABA receptor expression in the brain and reduce depression-like behaviours in animal models (Liwinski et al., 2023).
2. Animal studies also support that GABA signalling can be regulated by gut microbiota through vagal nerve pathways, further reinforcing this gut–brain interaction (Radjabzadeh et al., 2022).

Clinical Evidence:

1. GABA-producing bacteria such as *Parabacteroides*, *Eubacterium*, *Lactobacillus*, *Bifidobacterium*, *Bacteroides*, and *Blautia* (mostly *Bactroides fragilis*) have been associated with depression in humans (Gao et al., 2023; Mhanna et al., 2024).
2. Some taxa, including *Eggerthella*, Subdoligranulum, *Coprococcus*, and *Rumimococcaceae*, have been associated with reduced GABA production and depression in human populations (Radjabzadeh et al., 2022).

### 6.2 Glutamate-producing microbes

Glutamate, the main excitatory neurotransmitter in the brain, is vital for synaptic plasticity, cognition, and emotional regulation. Its dysregulation has been implicated in the pathogenesis of depression. Gut microbes may influence glutamate production and associated neuroinflammatory pathways:

Preclinical Evidence:

1. Some strains of bacteria can alter neurotransmitter production and transport across and the blood-brain barrier, which can affect neuronal signalling (Radjabzadeh et al., 2022).
2. SCFAs such as butyrate have been associated with neuroprotective effects and antidepressant-like behaviours, primarily through their effects on glutamate release in experimental animals (Yang et al., 2023).

Clinical Evidence:

1. Individuals with MDD often exhibit a reduced presence of microbial genes involved in glutamate biosynthesis (Kovtun et al., 2022).
2. Species such as *Faecalibacterium prausnitzii*, *Roseburia hominis*, and *Roseburia intestinalis* which contribute to glutamate and SCFA metabolism are typically reduced in depression (Kovtun et al., 2022; Gruenbaum et al., 2024).
3. Increases in *E. coli*, *Ruthenibacterium lactatiformans*, *Sellimonas*, *Eggerthella*, *Lachnoclostridium*, and *Hungatella* have been associated with greater depressive symptomatology (Radjabzadeh et al., 2022; Averina et al., 2024).

### 6.3 Serotonin-producing microbes

Serotonin (5-HT), a key neurotransmitter in mood regulation, is predominantly synthesized (∼95%) in the gut. Its availability is tightly linked to gut microbiota, particularly through modulation of tryptophan metabolism and SCFA production (Lukić et al., 2022).

Preclinical Evidence:

1. Microbial metabolites such as phenolic and indolic compounds stimulate enteric serotonin production, which can influence central serotonin levels through the vagus nerve or systemic circulation (Margoob et al., 2024).
2. *Turicibacter sanguinis* can stimulate intestinal serotonin production and has been shown to absorb serotonin via a mechanism similar to the mammalian serotonin transporter (SERT). This uptake is inhibited by fluoxetine (an SSRI), suggesting functional relevance to antidepressant pathways (Hoffman and Margolis, 2020).

Clinical Evidence:

1. *Lactobacillus* and *Bifidobacterium* are known to enhance serotonin synthesis and have been linked to reduced depressive symptoms, highlighting their potential probiotic applications (Kosyra et al., 2024).
2. Individuals with depression have significantly altered gut microbiota composition, indicating that tryptophan-derived metabolites are impacted by gut microbiota diversity (Aleti et al., 2022).

## 7 Summary of microbial taxa associated with depression

Multiple microbial taxa have been implicated in depression through their roles in neurotransmitter production, inflammation regulation, and GBA (Table 3). These include:

• Butyrate-producing taxa: *Faecalibacterium*, *Butyricimonas*, *Coprococcus*, *Subdoligranulum, E. ventriosum*, *Ruminococcus gauvreauii* group.
• GABA-related taxa: *L. rhamnosus*, *Bacteroides fragilis*, *Parabacteroides*, *Blautia*.
• Glutamate-modulating taxa: *F. prausnitzii*, *Roseburia* spp., *Sellimonas*, *Eggerthella*, *Hungatella.*
• Serotonin-modulating taxa: *Lactobacillus*, *Bifidobacterium*, *T. sanguinis*.
• Pro-inflammatory taxa linked to depressive symptoms: *Eggerthella*, *Desulfovibrio*, Enterobacteriaceae, *Lachnoclostridium.*

**TABLE 3:** A summary of the impact of different microbial taxa on depression through neurotransmitter and metabolite modulations.

Microbe	Phylum	Neurotransmitters & metabolites	Impact on depression
High impact on depression			
*Eggerthella spp.* (Mhanna et al., 2024)	Actinobacteria (Mhanna et al., 2024)	Linked to lower butyrate levels (Mhanna et al., 2024)	↑ Depression risk, pro-inflammatory (Mhanna et al., 2024)
*Actinomyces spp.* (Gao et al., 2023)	Actinobacteria (Gao et al., 2023)	Correlates with reduced butyrate-producing bacteria (Gao et al., 2023)	↑ Depression risk (Gao et al., 2023)
*Enterobacteriaceae spp.* (Gao et al., 2023)	Proteobacteria (Gao et al., 2023)	Produces lipopolysaccharides (LPS), leads to neuroinflammation (Gao et al., 2023)	↑ Depression risk (Gao et al., 2023)
*Desulfovibrio spp.* (Flynn et al., 2025)	Proteobacteria (Flynn et al., 2025)	Proinflammatory, associated with gut dysbiosis (Flynn et al., 2025)	↑ Depression risk (Flynn et al., 2025)
*Sellimonas spp.* (Radjabzadeh et al., 2022)	Firmicutes (Radjabzadeh et al., 2022)	Glutamate synthesis (Radjabzadeh et al., 2022)	↑ Depression risk (Radjabzadeh et al., 2022)
*Lachnoclostridium spp.* (Radjabzadeh et al., 2022)	Firmicutes (Radjabzadeh et al., 2022)	Glutamate and butyrate metabolism (Radjabzadeh et al., 2022)	↑ Depression risk (Radjabzadeh et al., 2022)
*Hungatella spp.* (Averina et al., 2024)	Firmicutes (Averina et al., 2024)	Butyrate and glutamate metabolism (Averina et al., 2024)	↑ Depression risk (Averina et al., 2024)
Low impact on depression			
*Faecalibacterium spp.* (Louis and Flint, 2009)	Firmicutes (Louis and Flint, 2009)	Major butyrate producer, anti-inflammatory (Louis and Flint, 2009)	↓ Depression risk, gut homeostasis (Louis and Flint, 2009)
*Coprococcus spp.* (Radjabzadeh et al., 2022)	Firmicutes (Radjabzadeh et al., 2022)	Produces butyrate, involved in neurotransmitter balance (Radjabzadeh et al., 2022)	↓ Depression risk (Radjabzadeh et al., 2022)
*Dialister spp.* (Radjabzadeh et al., 2022)	Firmicutes (Radjabzadeh et al., 2022)	SCFA production (butyrate) (Radjabzadeh et al., 2022)	↓ Depression risk (Radjabzadeh et al., 2022)
*Lactobacillus rhamnosus* (Liwinski et al., 2023)	Firmicutes (Liwinski et al., 2023)	Produces GABA, alters GABA receptor expression (Liwinski et al., 2023)	↓ Anxiety, ↓ Depression (Liwinski et al., 2023)
*Bifidobacterium spp.* (Kosyra et al., 2024)	Actinobacteria (Kosyra et al., 2024)	Produces serotonin and dopamine (Kosyra et al., 2024)	↓ Depression risk, mood regulation (Kosyra et al., 2024)
*Bacteroides fragilis* (Mhanna et al., 2024)	Bacteroidetes (Mhanna et al., 2024)	GABA synthesis, modulates gut-brain axis (Mhanna et al., 2024)	↓ Anxiety, ↓ Depression (Mhanna et al., 2024)
*Eubacterium spp.* (Gao et al., 2023)	Firmicutes (Gao et al., 2023)	GABA production (Gao et al., 2023)	↓ Depression risk (Gao et al., 2023)
*Subdoligranulum spp.* (Radjabzadeh et al., 2022)	Firmicutes (Radjabzadeh et al., 2022)	Butyrate producer, involved in neurotransmitter synthesis (Radjabzadeh et al., 2022)	↓ Depression risk (Radjabzadeh et al., 2022)
*Roseburia hominis* (Kovtun et al., 2022)	Firmicutes (Kovtun et al., 2022)	SCFA (butyrate) production (Kovtun et al., 2022)	↓ Depression risk (Kovtun et al., 2022)
*Ruminococcaceae family* (Radjabzadeh et al., 2022)	Firmicutes (Radjabzadeh et al., 2022)	Involved in glutamate and butyrate metabolism (Radjabzadeh et al., 2022)	↓ Depression risk (Radjabzadeh et al., 2022)
*Turicibacter sanguinis* (Hoffman and Margolis, 2020)	Firmicutes (Hoffman and Margolis, 2020)	Influences serotonin synthesis (Hoffman and Margolis, 2020)	↓ Depression risk, gut-brain signaling (Hoffman and Margolis, 2020)

↓ represent reduced/decreased; ↑ represent increased. GABA, gamma-aminobutyric acid; SCFA, short-chain fatty acids.

These associations reflect the dynamic and multifactorial nature of gut-brain interactions and suggest potential biomarkers or therapeutic targets for microbiota-based interventions in depression.

## 8 Positive and negative impacts of psychotropic medications on gut microbiota

The interaction of psychotropic drugs and gut microbiota has increased attention in current research highlights, with conflicting findings between positive and negative for microbiota composition and health.

### 8.1 Antidepressant class

Selective Serotonin Reuptake Inhibitors (SSRIs), a widely used class of antidepressants, exemplify this negative uncertainty through their variable effects on the gut microbiota. Several studies indicates that SSRIs potentially reduce alpha diversity and affect healthy microbial communities which could cause dysbiosis and low-grade inflammation. Sertraline and fluoxetine, for an example, also possess antibacterial action, disrupting both Gram-positive and Gram-negative bacteria, where evidence suggests that these changes could worsen gut barrier dysfunction and immunological activation in certain cases (Hatamnejad et al., 2022). For instance, Zhang W. et al. (2021), noted that certain antidepressant like fluoxetine may alter intestinal microbiota and compromise gut microbiome function which can adversely impact in certain situations particularly when gut microbiome is in a vulnerable state (Zhang W. et al., 2021). Research also suggests that SSRIs may lead to dysbiosis and inflammation in the stomach due to their antibacterial capabilities, which potentially limit the beneficial microbial species (Ramsteijn et al., 2020; Desorcy‐Scherer et al., 2024).

Alternatively, vortioxetine has gained prominence in the treatment of mood disorders due to its multimodal mode of action, which including serotonin reuptake inhibition and interactions with multiple serotonergic receptors. Vortioxetine’s action as a serotonin reuptake inhibitor and agonist at 5HT1A receptors is notable, as it acts as an antagonist at 5HT3 and 5HT7 receptors. Specifically, the 5HT7 receptor has been implicated in mood regulation and cognitive function. Such receptor-specific modulation is thought to facilitate neurogenesis and improve cognitive flexibility, which may address both emotional and cognition aspects of symptoms of depression. Vortioxetine’s broader physiological effects may extend to the GBA, which potentially supporting microbiome balance and overall health (Krupa et al., 2023). Besides that, Ye et al. (2021), also mentioned that certain genera such as *Lachnospira* and *Roseburia*, had a negative correlation to depression severity. This data demonstrates that Vortioxetine may induce a microbiome profile associated with better mental health outcomes. The GBA connecting the gut microbiota and the CNS is likely to be fundamental in mediating these effects (Ye et al., 2021).

### 8.2 Antipsychotic class

Antipsychotic drugs, particularly atypical antipsychotics including olanzapine and risperidone, are associated with serious metabolic health outcomes primarily though the alteration of metabolic pathways and altering gut microbiota. Evidence suggests that medication such as olanzapine drive the reduction of microbial richness in the gut, which correlates with adverse metabolic regulation outcomes, including weight gain and the risk for developing metabolic syndrome (Kamath et al., 2024; Pillinger et al., 2020; Jarari et al., 2025). Olanzapine, in particular, has demonstrated a substantial effect on consistent weight gain and the alteration of gut microbial profiles, causing a decrease in the Shannon’s index and beta diversity, subsequently affecting normal indexes of microbial diversity (Kamath et al., 2024).

Notably, lurasidone is an atypical antipsychotic, but has a different metabolic effect. Studies suggest lurasidone might help in developing better microbial diversity and has been called “gut neutral,” in contrast to the negative impacts of olanzapine and risperidone (Kamath et al., 2024; Collins et al., 2024). Preclinical studies suggest that Lurasidone enhances gut-derived metabolic profiles, making it a potentially promising treatment option for individuals at risk of metabolic side effects related to antipsychotic medications (Kamath et al., 2024; Munawar et al., 2021). Table 4 summarizes the classes of psychotropic medications, specifically contrasting antidepressants and antipsychotics, along with their mechanisms, microbiota impacts.

**TABLE 4:** Classes of psychotropic medications, contrasting antidepressants and antipsychotics.

Drug	Exemplars	Mechanism of action	Reported microbiota impacts	Key references
Antidepressant class				
SSRIs	Sertraline, Fluoxetine	Block SERT, increase synaptic 5-HT	Ambiguous effects: antimicrobial action; reduced alpha diversity; dysbiosis and pro-inflammatory changes possible in some contexts; microbial shifts linked to treatment response	Ramsteijn et al. (2020), Zhang et al. (2021a), Hatamnejad et al. (2022)
Multimodal	Vortioxetine	SERT inhibition + 5-HT receptor modulation (5-HT1A agonist, 5-HT3/5-HT7 antagonist)	Preliminary reports show increased microbial diversity and beneficial shifts (e.g., *Lachnospira*, *Roseburia*) associated with symptom improvement; 5-HT7 blockade proposed as key mechanism	Krupa et al. (2023), Ye et al. (2021)
Antipsychotic Class				
Atypical antipsychotics	Olanzapine, Risperidone	D2 antagonism + 5-HT receptor modulation	Consistently reduce microbial diversity (low Shannon’s index, altered β-diversity), linked to metabolic dysregulation (weight gain, insulin resistance)	Kamath et al. (2024), Pillinger et al. (2020), Jarari et al. (2025)
Atypical antipsychotic	Lurasidone	D2 antagonism + 5-HT7 antagonism	Preclinical/clinical evidence suggests neutrality or improvement in microbiota composition; may enhance gut-derived metabolic profiles, lower metabolic risk	Kamath et al. (2024), Collins et al. (2024), Munawar et al. (2021)

## 9 Demographic differences in gut microbiota and depression

Recent research has increasingly focused on the complex interplay between gut microbiota, demographic factors (such as sex and age), and MDD. Given the well-established sex-based disparities in the prevalence and presentation of MDD, emerging evidence suggests that differences in gut microbiota composition may partly explain these disparities.

Several studies have reported significant alterations in the gut microbiota of individuals with MDD, a condition known as dysbiosis. A comprehensive scoping review identified five studies reporting significant sex-specific differences in both alpha and beta diversity of gut microbiota among MDD patients compared to healthy controls (Niemela et al., 2024). Specifically, certain bacterial taxa appear to be more prevalent in women than in men. For example, female patients with MDD show higher relative abundances of specific bacteria compared to male patients under similar conditions (Niemela et al., 2024). In particular, women tend to have elevated levels of Actinobacteria, whereas men with MDD display lower levels of *Bacteroidetes* (Chen et al., 2018). Moreover, the severity of depressive symptoms has been linked to unique microbial signatures in each sex, suggesting that sex-specific biological processes may mediate the gut-brain connection in mood disorders (Niemela et al., 2024; Hu et al., 2023).

These findings underscore the importance of incorporating sex-stratified analyses in both the diagnosis and treatment of MDD. Gut microbial markers may hold potential as sex-specific diagnostic tools or treatment targets, highlighting the need for personalized approaches in mental health interventions (Niemela et al., 2024). In addition to sex differences, age-related changes in gut microbiota also appear to influence MDD pathogenesis. Notably, young adults (18–29 years) with MDD exhibit lower levels of *Firmicutes* and higher levels of *Bacteroidetes*, while middle-aged adults (30–59 years) demonstrate the opposite trend (Chen et al., 2020). In a study of young adults with depression, specific taxa such as *Neisseria spp*. and *Prevotella nigrescens* were found in significantly greater abundance among depressed individuals compared to healthy controls (Wingfield et al., 2021).

Furthermore, alterations were observed in the oral microbiome, with 21 bacterial species exhibiting significant differences in abundance, suggesting a broader microbial dysregulation involving both oral and gut environments (Wingfield et al., 2021). These microbial patterns may reflect underlying inflammatory mechanisms or contribute directly to the etiology of mood disorders (Kerff et al., 2024). In older adults, particularly those experiencing late-life depression, distinct microbial signatures have also been identified. Although taxonomic changes in this population are less well-defined, certain microbial alterations have been linked to both gastrointestinal symptoms and depressive mood (Miyaho et al., 2022). A common finding among elderly individuals is a decline in beneficial bacteria, such as *Lactobacillus* and *Bifidobacterium*, which has been associated with increased vulnerability to depression (Kumar et al., 2023). These age-related shifts may be influenced by cumulative health issues, diet, medication use, and systemic inflammation, all of which can affect gut-brain axis functioning (Zhang Q. et al., 2021).

Beyond age and sex, body mass index (BMI) and lifestyle-related factors such as ethnicity and urbanization also appear to shape gut microbiota composition. Higher BMI has been associated with reduced microbial diversity, a change that may contribute to both metabolic dysfunction and altered gut-brain communication (Cryan et al., 2019). Ethnicity, which often correlates with specific dietary and cultural practices, has also been linked to distinct gut microbial profiles, reflecting differences in environmental exposures and genetic backgrounds (Ren et al., 2023). Similarly, living environment urban versus rural plays a significant role. Urbanization is associated with reduced intra-individual gut microbiota diversity and increased variability between individuals, likely due to changes in diet, stress levels, antibiotic exposure, and environmental microbiota contact (Ren et al., 2023).

Taken together, these findings illustrate the critical role of demographic factors in shaping gut microbial ecosystems and influencing the onset, severity, and manifestation of depression. Recognizing and accounting for these variables is essential in developing more targeted, personalized strategies for mental health assessment and treatment.

## 10 Lifestyle and behavioral considerations in GBA modulation

Lifestyle factors including high-fibre diets, fermented foods, and prebiotics can promote microbiome diversity and may complement psychobiotic treatments by supporting mood and resilience to stress (Appleton, 2018; Aziz et al., 2024; Leeuwendaal et al., 2022). While behavioural frameworks and cultural tailing such as age, gender, ethnicity, surroundings) may impact adherence, these factors are less significant than the pharmacological modulation which is the focus of this research. Broader measures, such as microbiome-informed food policy or educational campaigns, may help to increase adoption, although they are only addressed here to offer context for psychobiotic therapies.

## 11 Research gaps and limitations

Nonetheless, despite progress we have barriers that inhibit GBA findings integrated into clinical practice. Human based studies often utilize small number of subjects or lack demographic diversity, and microbiota and targeted therapies are variable in strain, dosing and duration. This variability limits the comparability of studies, and we lack standardisation of microbial characterisation and classification of our intervention. Most studies have identified correlation rather than causation and we have unknown confounders, for example nutrient composition, treatment or regimens, sleep and socio-economic status.

Importantly, comprehensive strain-dose-duration-specific data are inconsistently recorded and hence cannot be provided systematically in this study. This omission emphasises are critical need that future research must fill. Large, well-controlled, and demographically varied cohorts, together with standardised methods and multi-omics techniques, are required to elucidate pathways and improve clinical translation.

## 12 Future directions and clinical translation

The next hurdle in the research of GBA is to turn foundational insights into functional therapeutics. Microbiome-based biomarkers could allow earlier identification and monitoring of potential therapies in depression, but repeatability is an issue due to changing effects of diet, medications and host physiology (Sochacka et al., 2024). Personalised microbiome-targeted therapeutics using custom probiotics, prebiotics, and synbiotics, are promising, but need to be thoroughly assessed on causative mechanisms, strain specific efficacy, and long-term safety (Ma et al., 2023). Regulatory conditions and quality assurance mechanisms are similarly even more important.

Psychobiotics with established mental health benefits are emerging as adjunct therapies to standard psychiatric treatments, but widespread application will require evidence of reliable efficacy and safety. The adoption of these ideas into clinical practice will require standardized methods of protocols, longitudinal assessments, and equitable availability.

Ultimately, improving clinical translation will require not just technical innovation but also collaborative, multidisciplinary work that links neurology with psychiatry. Gastrointestinal, dietic science and behavioural health. By bridging these disciplines, the field is ready to make advances with new, individualized solutions for depression prevention and management.

## 13 Conclusion

GBA represents a promising frontier in understanding and managing MDD. Emerging evidence underscores the role of gut microbiota in modulating mood through neuroimmune pathways, neurotransmitter production, and HPA axis regulation. Microbial imbalances are consistently linked to depressive symptoms, suggesting that restoring gut health may offer novel therapeutic opportunities. Microbiota-targeted strategies such as probiotics, prebiotics, and dietary interventions show potential as adjuncts to conventional treatments. However, progress is limited by small sample sizes, methodological inconsistencies, and a lack of causal evidence. Future research should focus on identifying reliable microbial biomarkers, leveraging advanced omics technologies, and developing personalized, evidence-based interventions. Integrating gut-focused approaches with existing therapies may enhance treatment outcomes, reduce resistance, and support holistic mental healthcare. Addressing regulatory, ethical, and implementation challenges will be essential to translating gut-brain research into effective, accessible clinical practice. Future research should prioritize human randomized controlled trials to validate microbial biomarkers (e.g., *Coprococcus* abundance) and explore pharmacomicrobiomics how gut microbes modulate drug pharmacokinetics and pharmacodynamics in depression.
